# Patterns of overall mortality by race/ethnicity and socioeconomic status in insured cancer patients in Southern California

**DOI:** 10.1007/s10552-021-01414-4

**Published:** 2021-03-30

**Authors:** Robert M. Cooper, Joanie Chung, Tiffany Hogan, Reina Haque

**Affiliations:** 1 Department of Pediatric Oncology, Hematology/Oncology, Kaiser Permanente Los Angeles Medical Center, 4967 Sunset Blvd, Los Angeles, CA USA; 2 Department of Research & Evaluation, Kaiser Permanente Southern California, 100 S. Los Robles, Pasadena, CA USA; 3 Department of Health Systems Science, Kaiser Permanente Bernard J. Tyson School of Medicine, 99 S. Los Robles, Pasadena, CA USA

**Keywords:** Cancer outcomes, Health disparity, Mortality, Race, Ethnicity, Socioeconomic status

## Abstract

**Purpose:**

We evaluated the influence of race/ethnicity and geocoded socioeconomic status (SES) on all-cause mortality in cancer patients with health insurance.

**Methods:**

We identified adults diagnosed with eight common cancers from 2009 to 2014 from the California Cancer Registry and followed them through 2017 (8 years maximum). We calculated person-year mortality rates by race/ethnicity and SES. Adjusted hazard ratios for the association between overall mortality and race/ethnicity and SES were estimated using Cox proportional hazards models accounting for other demographics, stage at diagnosis, and cancer treatments.

**Results:**

A total of 164,197 adults were diagnosed with cancer originating from breast, prostate, lung, colon, skin melanoma, uterus, kidney, and bladder. For all race/ethnic groups combined, the mortality rates from lowest to highest SES groups were 112.1/1000 PY (lowest); 100.2/1000 PY (lower-middle); 91.2/1000 PY (middle); 79.1/1000 PY (upper-middle); and 63.5/1000 PY (upper). These rates suggest that person with lowest SES have a markedly increased mortality risk after cancer diagnosis even if they have health insurance. In multivariable analyses, those in the lowest SES group had a 40–78% increased risk of all-cause mortality compared to those in the upper SES group across all race/ethnicities. For example, within African Americans, the adjusted mortality risk was up to 61% higher (HR 1.61, 95% CI 1.41–1.83) in the lowest SES group compared to the highest SES group.

**Conclusion:**

This study suggests disparities in overall mortality risk after cancer diagnoses persist even in a cohort with health insurance, and that SES is an important driver of this disparity.

## Introduction

In the U.S., the cancer incidence rate was 442.2/100,000 (based on 2013–2017 cases) and the cancer mortality rate was 158.3/100,000 (2013–2017). In California, the cancer incidence was 404.8/100,000 and 142.4/100,000 for the similar time periods [1]. Disparities in mortality by race/ethnicity in patients with cancer have been well documented [2–6]. Many different factors contribute to these disparities including baseline socioeconomic (SES); distributions by race; access to screening and care; insurance status; tumor biology and characteristics; and differences in disease management and care delivery in various healthcare settings. Having health insurance is strongly associated with better overall survival as well as cancer-specific outcomes such as recurrence and progression-free survival [5–13]. Although several recent studies have examined cancer outcomes by both race/ethnicity and SES, they included both patients with and without health insurance, making it difficult to disentangle the effects of health insurance coverage from other SES factors. Given this gap, our goal was to evaluate the influence of both race/ethnicity and SES as well as the intersection of these two factors on all-cause mortality in an insured population of diverse adult cancer patients diagnosed in southern California between 2009 and 2014 and followed through 2017.

## Methods

### Subjects

We identified adults (≥ 20 years) diagnosed with eight common cancers (breast, prostate, lung, colon, melanoma, endometrium, kidney, and bladder) from 1 January 2009 through 31 December 2014 from the California Cancer Registry (CCR) who lived in the these southern California counties: Imperial, Los Angeles, Orange, Riverside, San Bernardino, and San Diego. Additional inclusion criteria included affirmative pathological cancer diagnosis at invasive stages (American Joint Committee on Cancer TNM stages I–IV) and confirmed health insurance coverage at the time of diagnosis. Health coverage included managed care (health maintenance organization [HMO]; preferred provider organization [PPO]; other managed care); Medicare; Medicaid; dual covered (Medicare and Medicaid); and other private insurance. The study was approved by internal review boards (IRBs) of two institutes, Kaiser Permanente and State of California, and both waived the requirement for written or verbal consent due to its exclusive use of de-identified data.

### Main outcomes and variables

The main outcome of interest was all-cause (overall) mortality. Patients’ records were followed through 31 December 2017 regarding vital status. Date of deaths and all variables were also ascertained from the CCR. Main variables of interest included race/ethnicity (non-Hispanic White; African American/Black; Hispanic; Asian/Pacific Islander; Native American; Other/Mixed) coded according to the North American Association of Central Cancer Registries algorithm [2] and SES based on U.S. 2010 Census data. Because we did not have addresses for subjects in this de-identified dataset, we used the geocoded SES variable that was available from the CCR. This geocoded SES variable is a composite of seven SES indicators from the U.S. 2010 census data at the block level (education; median household income; percentage living 200% below poverty level; percentage of blue collar workers; percentage older than 15 years in workforce without jobs; median rent; and median house value) [14]. The methodology how these factors were used to derive a composite SES score for each census block and categorized into quintiles (Q1: lowest; Q2: lower-middle; Q3: middle; Q4: upper-middle; and Q5: highest) based on the statewide distribution is described by Yost et al. [14] and has been used in prior studies [15, 16]. Covariates we extracted included age and stage at diagnosis; sex; year of diagnosis; anatomic cancer site; primary cancer therapy (surgery); adjuvant therapy (receipt of chemotherapy, radiation, and hormonal); health insurance source; and county of residence.

### Statistical analysis

Because patients had varying follow-up lengths based on time of diagnosis, we calculated person-year (PY) mortality rates and 95% confidence intervals by and race/ethnicity and SES. Multivariable-adjusted hazard ratios (HR) for the association between all-cause mortality and race/ethnicity and SES were estimated using Cox proportional hazards models accounting for the aforementioned covariates.

## Results

This cohort of insured patients was followed for a maximum of 8 years. A total of 164,197 adults were diagnosed with cancers of the breast, prostate, lung, colon, melanoma, uterus, kidney, and bladder in southern California; the total of numbers of deaths by the end of the data analysis period was *n* = 41,727. Table 1 shows the distribution of the patients by demographics, health insurance payer, and tumor characteristics. About 55.5% of the patients were female, and roughly half (48.9%) of the patients were over age 65 years. Patients were covered through various health insurance plans, with the majority belonging to managed care programs (HMO, PPO, other private insurance, 70.9%), while a quarter were covered through Medicare (25.9%). Small percentages of patients received insurance through Medicaid (0.1%); both Medicare and Medicaid (2.6%); or other low-income programs such as county-based safety net insurance (0.4%). Based on geocoded information, 11.3% were in the lowest SES group; 16.8% in lower-middle; 20.4% middle; 24.4% in upper-middle; and 27.2% in the upper income group. The cohort was diverse with 63.5% non-Hispanic Whites; 7.8% African Americans/Blacks; 18.1% Hispanics; 9.3% Asian/Pacific Islanders (PI); 0.3% Native American; and 1.0% of other/mixed/unknown race/ethnicity. Breast (30.0%) and prostate cancers (20.7%) accounted for half of the cancers diagnosed. Overall, 81.4% of these eight common cancers were diagnosed at early stages (AJCC TNM I–III). While 74.3% of patients underwent surgery as primary therapy, the percentage of those who had adjuvant chemotherapy (22.7%), radiation (22.5%), or hormonal (14.7%) was lower.

**Table 1:** Demographic and tumor characteristics of cancer cases diagnosed in 2009–2014

	Total	
	*n*	%
Total	164,197	100
Age at diagnosis (years)		
20–39	5,735	3.5
40–64	78,062	47.5
65 +	80,400	49.0
Sex		
Male	73,085	44.5
Female	91,100	55.5
Transsexual/transgender/other	4	0.002
Year of diagnosis		
2009–2010	56,205	34.2
2011–2012	53,992	32.9
2013–2014	54,000	32.9
Health insurance		
Managed care (HMO/PPO/managed care)	116,548	71.0
Other private	702	0.4
Medicare	42,557	25.9
Medicaid	4,189	2.6
Dual eligible (Medicare + Medicaid)	201	0.1
Socioeconomic status		
Lowest	18,513	11.3
Lower-middle	27,506	16.8
Middle	33,480	20.4
Upper-middle	40,001	24.4
Highest	44,697	27.2
Race/ethnicity		
White, non-Hispanic	104,314	63.5
African American/Black	12,789	7.8
Hispanic	29,713	18.1
Asian/PI	15,269	9.3
Native American	473	0.3
Other/unknown	1,639	1.0
County of residence		
Imperial	588	0.4
Los Angeles	75,743	46.1
Orange	28,012	17.1
Riverside	18,112	11.0
San Bernardino	14,558	8.9
San Diego	27,184	16.6
Major anatomical site		
Breast	49,398	30.1
Prostate	33,999	20.7
Lung	23,970	14.6
Colon	16,646	10.1
Melanoma (skin)	14,614	8.9
Endometrium	10,594	6.5
Kidney and renal	9,259	5.6
Bladder	5,717	3.5
Stage at diagnosis (AJCC TNM)		
I	64,391	39.2
II	47,444	28.9
III	21,857	13.3
IV	22,631	13.8
Unknown	7,874	4.8
Location		
Localized	99,476	60.6
Regional	38,679	23.6
Remote	23,451	14.3
Unknown	2,591	1.6
Primary therapy (surgery)		
No	42,218	25.7
Yes	121,928	74.3
Unknown	51	0.03
Chemotherapy		
No	124,719	76.0
Yes	37,255	22.7
Unknown	2,223	1.3
Radiation		
No	127,084	77.4
Yes	36,992	22.5
Unknown	121	0.07
Hormone therapy		
No	136,244	82.9
Yes	24,051	14.7
Unknown	3,902	2.4

Table 2 shows the person-year (PY) mortality rates stratified by race/ethnicity and SES. The highest mortality rates were observed in the lowest SES groups compared to patients in the highest SES group; this pattern persisted across all race/ethnic groups. For all race/ethnic groups combined, the mortality rates from lowest to highest SES groups were 112.1/1000 PY (lowest); 100.2/1000 PY (lower-middle); 91.2/1000 PY (middle); 79.1/1000 PY (upper-middle); and 63.5/1000 PY (upper). This corresponds to a 70% increased risk (crude HR 1.70, 95% CI 1.65–1.72) of all-cause mortality when comparing the those in the lowest SES group versus the upper group, suggesting that lower SES is markedly related to risk of death after cancer diagnosis, even if both groups have health insurance.

**Table 2:** Overall mortality rates by SES quintiles and race/ethnicity

Socioeconomic status#	Lowest		Lower-middle		Middle		Upper-middle		Highest	
	# deaths	Rate per 1,000 PY (95% CI)	# deaths	Rate per 1,000 PY (95% CI)	# deaths	Rate per 1,000 PY (95% CI)	# deaths	Rate per 1,000 PY (95% CI)	# deaths	Rate per 1,000 PY (95% CI)
Race/ethnicity										
White, non-Hispanic	2,624	141.1 (135.8, 146.6)	4,718	118.8 (115.4, 122.2)	6,328	102.0 (99.5, 104.5)	7,439	86.5 (84.6, 88.5)	7,563	66.7 (65.2, 68.2)
African American/Black	1,135	131.4 (123.8, 139.2)	980	105.3 (98.8, 112.1)	746	95.0 (88.3, 102.0)	541	72.7 (66.7, 79.1)	285	65.5 (58.1, 73.6)
Hispanic	1,664	80.6 (76.7, 84.5)	1,627	73.3 (69.8, 77.0)	1,317	67.3 (63.7, 71.0)	954	60.6 (56.8, 64.6)	517	49.9 (45.7, 54.4)
Asian/PI	311	103.5 (92.3, 115.7)	626	85.0 (78.5, 92.0)	707	75.5 (70.0, 81.3)	771	62.8 (58.4, 67.4)	716	55.0 (51.0, 59.2)
Native American	21	133.6 (82.7, 204.3)	24	70.2 (45.0, 104.4)	26	76.1 (49.7, 111.5)	30	119.7 (80.8, 170.9)	20	68.9 (42.1, 106.4)
Other/mixed/unknown	5	13.7 (4.5, 32.1)	4	5.5 (1.5, 14.2)	10	10.5 (5.0, 19.3)	9	5.9 (2.7, 11.2)	9	4.4 (2.0, 8.3)

Table 3 shows the association between SES and overall mortality stratified by race/ethnicity. The multivariable-adjusted HRs accounted for all the variables listed in Tables 1 and 2, including demographics, health insurance source, county of residence, anatomic cancer site, stage, and cancer treatments. Consistent with the person-year mortality rates, the adjusted HRs were greater in each of the lower SES groups as compared to the highest SES group (the reference) in all race/ethic groups. Interestingly, the disparity in all-cause mortality by SES was most pronounced in non-Hispanic Whites. Non-Hispanic Whites in the lowest SES group had a 78% increased risk of mortality (adjusted HR 1.78, 95% CI 1.70–1.87) than Whites in the highest SES group (reference). In African Americans/Blacks, the adjusted mortality risk varied from 5% higher (adjusted HR 1.05, 95% CI 0.91–1.22 in upper-middle) to 61% higher (adjusted HR 1.61, 95% CI 1.41–1.83) in the lowest SES groups compared to the highest SES group. Such disparity in mortality risk was less apparent in Hispanics which varied from 22% higher in the upper-middle group (adjusted HR 1.22, 95% CI 1.09–1.36) to 40% higher in the lowest SES group (adjusted HR 1.40, 95% CI 1.26–1.54) compared with the highest SES group. We could not robustly calculate the HRs for Native Americans (11 deaths) nor for those in the Other/Mixed/Unknown (three deaths) group given the low numbers of deaths.

**Table 3:** HRs for all-cause mortality and socioeconomic status, stratified by race/ethnicity

Race/ethnicity	Crude		Adjusted	
	HR	95% CI	HR	95% CI
Non-Hispanic White				
Lowest	2.06	(1.97, 2.15)	1.78	(1.70, 1.87)
Lower-middle	1.74	(1.68, 1.80)	1.60	(1.54, 1.67)
Middle	1.51	(1.46, 1.56)	1.41	(1.36, 1.45)
Upper-middle	1.28	(1.24, 1.33)	1.26	(1.22, 1.30)
Highest	1.00	Ref	1.00	Ref
African American/Black				
Lowest	1.94	(1.71, 2.21)	1.61	(1.41, 1.83)
Lower-middle	1.57	(1.37, 1.79)	1.42	(1.24, 1.62)
Middle	1.43	(1.25, 1.64)	1.34	(1.17, 1.54)
Upper-middle	1.10	(0.96, 1.27)	1.05	(0.91, 1.22)
Highest	1.00	Ref	1.00	Ref
Hispanic				
Lowest	1.56	(1.41, 1.72)	1.40	(1.26, 1.54)
Lower-middle	1.43	(1.30, 1.58)	1.34	(1.21, 1.48)
Middle	1.33	(1.20, 1.47)	1.22	(1.11, 1.36)
Upper-middle	1.20	(1.08, 1.33)	1.22	(1.09, 1.36)
Highest	1.00	Ref	1.00	Ref
Asian/Pacific Islander				
Lowest	1.83	(1.60, 2.10)	1.57	(1.38, 1.81)
Lower-middle	1.53	(1.37, 1.70)	1.40	(1.26, 1.56)
Middle	1.35	(1.22, 1.50)	1.19	(1.07, 1.33)
Upper-middle	1.14	(1.03, 1.27)	1.09	(0.98, 1.21)
Highest	1.00	Ref	1.00	Ref

Adjusted HRs could not be robustly calculated for Native Americans and other/mixed/unknown groups due to missing variables

Table 4 shows the association between overall mortality and race/ethnic groups, stratified by SES. In these multivariable models, overall mortality risk was statistically significantly lower in Hispanics and Asian/Pacific Islanders compared with non-Hispanic Whites (reference group) in every SES group. For example, in the lowest SES group, mortality risk was 36% lower Hispanics (adjusted HR 0.64, 95% CI 0.60–0.68) and 22% lower in Asian/Pacific Islanders (adjusted HR 0.78, 95% CI 0.69–0.89) compared to Whites (reference group). Interestingly, although African Americans/Blacks had lower point estimates of adjusted HR compared to Whites, the 13% lower mortality risk (adjusted HR 0.87, 95% CI 0.80–0.96) in this group reached statistical significance among those in the upper-middle SES group. As in Table 3, we did not calculate the HRs for Native Americans and those in other/mixed/unknown group due to small numbers of deaths. Because the fraction of Whites and Asians/Pacific Islanders was skewed towards higher SES groups, we examined mortality risk by race/ethnicity after adjusting for SES and other covariates in a sensitivity analysis. As shown in Table 5, we found no difference in mortality risk between African Americans/Blacks (adjusted HR 1.02, 95% CI 0.98–1.05) compared to Whites, and a reduced mortality risk among Hispanics (adjusted HR 0.83, 95% CI 0.81–0.86) and Asians/Pacific Islanders (adjusted HR 0.75, 95% CI 0.73–0.78).

**Table 4:** Adjusted risk of all-cause mortality and race/ethnicity by SES

SES	Crude		Adjusted	
	HR	95% CI	HR	95% CI
Lowest				
Non-Hispanic White	1.00	Ref	1.00	Ref
African Amer/Black	0.92	(0.86, 0.98)	0.96	(0.89, 1.04)
Hispanic	0.57	(0.53, 0.60)	0.64	(0.60, 0.68)
Asian/Pacific Islander	0.72	(0.64, 0.81)	0.78	(0.69, 0.89)
Lower-middle				
Non-Hispanic White	1.00	Ref	1.00	Ref
African Amer/Black	0.88	(0.82, 0.94)	0.94	(0.87, 1.01)
Hispanic	0.62	(0.58, 0.65)	0.69	(0.65, 0.73)
Asian/Pacific Islander	0.71	(0.65, 0.77)	0.78	(0.71, 0.84)
Middle				
Non-Hispanic White	1.00	Ref	1.00	Ref
African Amer/Black	0.92	(0.86, 0.99)	0.99	(0.92, 1.07)
Hispanic	0.66	(0.62, 0.70)	0.71	(0.67, 0.75)
Asian/Pacific Islander	0.73	(0.67, 0.78)	0.76	(0.70, 0.82)
Upper-middle				
Non-Hispanic White	1.00	Ref	1.00	Ref
African American/Black	0.84	(0.77, 0.92)	0.87	(0.80, 0.96)
Hispanic	0.70	(0.65, 0.75)	0.79	(0.74, 0.85)
Asian/Pacific Islander	0.72	(0.67, 0.78)	0.78	(0.72, 0.84)
Highest				
Non-Hispanic White	1.00	Ref	1.00	Ref
African American/Black	0.98	(0.87, 1.09)	1.00	(0.89, 1.13)
Hispanic	0.75	(0.68, 0.82)	0.80	(0.74, 0.88)
Asian/Pacific Islander	0.81	(0.75, 0.88)	0.89	(0.83, 0.96)

Adjusted HRs could not be robustly calculated for Native Americans and other/mixed/unknown groups due to missing variables

**Table 5:** Adjusted mortality risk by race/ethnicity adjusted for SES and other factors

	Crude		Adjusted^a^	
	HR	95% CI	HR	95% CI
Non-Hispanic White	1.00	Ref	1.00	Ref
African American/Black	1.08	(1.04, 1.11)	1.02	(0.98, 1.05)
Hispanic	0.76	(0.74, 0.78)	0.83	(0.81, 0.86)
Asian/Pacific Islander	0.76	(0.73, 0.79)	0.75	(0.73, 0.78)

^a^Adjusted for SES; cancer site; age at diagnosis; stage; gender; year; healthcare setting (managed care or other private); county; receipt of surgery; receipt of adjuvant therapy (chemotherapy, radiation, hormonal)

## Discussion

Inadequate health insurance coverage is a major contributor to disparities in cancer outcomes. Other potential factors include tumor biology differences, health literacy, difficulties navigating the healthcare system, and providers’ implicit bias. Our study suggests disparities in overall mortality risk persist in patients with cancer, even in a cohort with health insurance coverage, and that lower SES is an important driver of this disparity; for example, the mortality risk was about 70% greater in those with lower SES than those with higher SES. When examining the person-year mortality rates for each race/ethnicity by SES for the eight most common cancers combined, a similar pattern emerged whereby mortality rates were higher in the lower SES versus the higher SES groups across all race/ethnicities. This association was also seen in multivariable-adjusted analyses that examined mortality risk by race/ethnicity after adjusting for SES (Table 5). Many factors might have contributed to the greater overall mortality risk in the lower SES groups. Such factors include lifestyle factors such as diet and smoking; health literacy; lower cancer screening utilization; environmental contaminants; and transportation issues affecting access to care that may lead to later cancer stage at presentation. A recent review of European literature noted that an increase in incidence of many cancer types was correlated with SES [17]. Clegg et al. evaluated the Surveillance, Epidemiology, and End Results (SEER) data and found that later stage at presentation in different cancer types was greater in those with lower SES [18]. Hence, SES has been found to be strongly associated with both cancer incidence and later stage at presentation. Additionally, during cancer therapy, patients in the lower SES groups might have experienced differences in access to treatments, care coordination, clinical trials, and providers’ implicit bias which may also relate to mortality [19–23]. However, the previous studies included a mixture of patients with and without health insurance; thus, it is not clear to what degree lower SES contributed to cancer outcome disparities in those studies. By contrast, in this present study that included only insured patients, we found that Hispanics and Asians/Pacific Islanders had lower overall mortality rates in each of the SES groups compared to non-Hispanic Whites and African American/Blacks (Table 2). Non-Hispanic Whites and African Americans/Blacks had similar mortality rates in each of the SES groups, except for the upper-middle and lower-middle groups, in which African Americans/Blacks had even lower mortality risk compared to non-Hispanic Whites. Of note, Hispanics had lower mortality rates in every SES group than non-Hispanic Whites, African Americans/Blacks, and Asian/PIs (Table 2). This observation is consistent with the well described, but poorly understood, “Hispanic Paradox” whereby Hispanics have lower mortality across many disease conditions compared to non-Hispanic Whites possibly due to beneficial genetic polymorphisms, diet, and cultural practices [24].

Our study found that disparities exist in overall mortality by SES in all race/ethnic groups. For example, in African Americans/Blacks, those in the lowest SES group had a 61% increased risk of death (adjusted HR 1.61, 95% CI 1.41, 1.83) compared to those in the highest SES group. The mortality disparity was the lowest within Hispanics, with those in the lowest SES group having a 40% increased mortality risk (adjusted HR 1.40, 95% CI 1.26–1.54) compared to those in the highest SES group. It is unclear why the mortality differences were greater among Whites when examining their risk by SES. Nonetheless, SES seems to be an important risk factor even after adjusting for important covariates. The observation that health disparities related to race/ethnicity and SES are not novel; however, less literature exists that seeks to parse out the reasons as to why patients with noted health disparities have poorer outcomes.

The majority of large cohort studies examining cancer outcomes have also used cancer registry data, which lack data on patient-level factors such as comorbidities present at the time of diagnosis [14–16, 18, 25, 26]. For example, it is possible that patients with lower SES have more comorbidities such as obesity, hypertension, depression, and diabetes or negative lifestyle factors (e.g., smoking). The presence and management of these conditions can influence the ability to deliver optimal cancer care. Thus, to uncover these potential contributing factors to cancer outcomes, it will be necessary to access the promise of big data provided by robust electronic medical systems. Since the passing of the Accountable Care Act in 2010, healthcare systems have rapidly instituted electronic medical records (EMR) and consolidated them into larger linked organizations. This technological advancement will provide an opportunity for future research to link patient-level comorbidity data with registry data to help address how the presence of chronic conditions affect cancer outcomes.

Another research opportunity will be to examine how healthcare system-level factors affect cancer outcomes in the context of health disparities. In 2013, the Institute of Medicine (IOM) published a framework of an effective cancer care delivery system which leverages an EMR, systematic quality measurement with performance improvement, robust safety nets, and use of tumor board review that can lead to accessible, affordable cancer care [13]. Optimal cancer care is complex, but integrated healthcare systems may provide advantages by facilitating care coordination compared to other systems of care. For example, the Veterans Administration, an integrated healthcare system, has reduced some of the racial health disparities in their population [27]. Further, a study reported that patients cared for in a National Cancer Institute Comprehensive Cancer Center (NCICCC) had better overall survival compared to those who received care in the community [19]; however, whether the longer survival was related to better treatments, better adherence to guidelines, or care coordination was not addressed in that study. Hence, integrated organizations may be poised to study the influence of care delivery systems on outcomes. Healthcare organizations can leverage cancer care coordinators and multidisciplinary tumor boards to help mitigate cancer care disparities.

Further, the influence of social determinants of health must be examined to improve the care of socially disadvantaged patients. Issues related to travel distance, access to transportation to cancer care facilities, access to telemedicine, and food insecurity may all be magnified during cancer care where optimal management becomes more complex. To potentially mitigate the effects of these social determinants, they first must be recognized by the medical providers. Once recognized, the medical systems must either implement programs to support patients or have partnerships with local community organizations to do so. All of this requires systems level support through social work, patient navigation, and cancer case management. Research into which actionable factors are essential to improve optimal and equitable care is needed.

This study has several strengths. First, we examined differences in mortality risk an insured population and, thus, we could evaluate the role of SES and race/ethnicity without the pronounced confounding effect of variable health insurance coverage. Second, this large population-based study is generalizable given that we captured data from the state’s cancer registry with decreased risk of selection or survivorship biases. Finally, the eight-year maximum follow-up ensured identification of adequate numbers of deaths to conduct robust analyses. For the most common cancers, cancer registries across the U.S. reported confirmation or detection rates of 95% or greater for death date and cause [28].

As with any observational study, certain limitations were present. As the CCR did not include important lifestyle data such as obesity, smoking, or physical activity, we could not adjust for these variables. We also did not have information on medical comorbidities. Patients in the lower SES groups might have had more comorbidities, and these conditions may be related to cancer treatment choices and their responses, both of which affect mortality risk. The study also relied on the state’s cancer registry categorization of race/ethnicity, which is not always completely accurate. However, prior reports have shown this misclassification to be relatively low [17, 18]. Another limitation of the study is related to ecologic fallacy, specifically, we used geocoded SES information that was available in the CCR because individual-SES information is not captured by the registry [14, 15]. Thus, it is possible that geocoded SES variable may not accurately represent individual-level SES [15]. Although we acknowledge that different cancers have different prognoses, the goal of this study was to understand the associations between SES and race/ethnicity and overall mortality in a large diverse population-based cohort of insured patients in California. Nonetheless, it is very possible that SES and race/ethnicity may have different influences on outcomes for individual cancers. Examining individual cancers and their outcomes was beyond scope of this work, although it is worthwhile direction for future analysis. Of note, other population-based studies using national cancer databases such as the CDC’s National Program of Cancer Registries (NPCR) and/or the NCI’s Surveillance, Epidemiology, and End Results (SEER) program have also used overall survival to examine trends [29]. Also, this report examined the eight most common cancers combined, without including the rare malignancies. Therefore, it is possible that mortality relating to other cancer types may have different interactions with race/ethnicity and SES. Despite these potential limitations, the study was based on a large and diverse population-based sample of patients.

In summary, in this first comprehensive analysis of a completely insured population of patients with new cancer diagnoses, we observed disparities in mortality risk suggesting that SES was more strongly linked to overall mortality than race/ethnicity. It is possible that insured cancer patients with lower SES may have more comorbidities, lower health literacy, transportation issues affecting access to cancer screening and early treatment, and less social support than wealthier patients that contribute to their increased risk. Healthcare providers’ implicit bias might have also contributed to higher mortality in lower SES group. It is also important to realize that having health insurance alone does not completely mitigate the well-recognized effects of socioeconomic status on health outcomes, especially for diseases with complex interdisciplinary management such as the malignancies that we studied. It is imperative for future studies to identify which specific potentially actionable factors lead to this observed health disparity.

## Data Availability

Per contract with the State of California, we cannot share the dataset; however, all investigators can apply directly to the California Cancer Registry to retrieve these data (https://www.ccrcal.org/retrieve-data/).

## Code availability

Programming codes or logic for the analysis can be requested by writing to the corresponding author.
